# Whole-brain MR spectroscopic imaging reveals regional metabolite abnormalities in perinatally HIV infected young adults

**DOI:** 10.3389/fnins.2023.1134867

**Published:** 2023-03-02

**Authors:** Teddy Salan, Elizabeth J. Willen, Anai Cuadra, Sulaiman Sheriff, Andrew A. Maudsley, Varan Govind

**Affiliations:** ^1^Department of Radiology, University of Miami Miller School of Medicine, Miami, FL, United States; ^2^Department of Pediatrics, University of Missouri-Kansas City School of Medicine, Kansas City, MO, United States; ^3^Department of Pediatrics, Mailman Center for Child Development, University of Miami Miller School of Medicine, Miami, FL, United States

**Keywords:** perinatal HIV, MRSI, brain, metabolites, young adults

## Abstract

Perinatally acquired HIV (PHIV) has been associated with brain structural and functional deficiencies, and with poorer cognitive performance despite the advent of antiretroviral therapy (ART). However, investigation of brain metabolite levels in PHIV measured by proton magnetic resonance spectroscopy (MRS) methods, is still limited with often inconclusive or contradictory findings. In general, these MRS-based methods have used a single voxel approach that can only evaluate metabolite concentrations in a few select brain anatomical regions. Additionally, most of the published data have been on children perinatally infected with HIV with only a few studies examining adult populations, though not exclusively. Therefore, this prospective and cross-sectional study aims to evaluate metabolite differences at the whole-brain level, using a unique whole-brain proton magnetic resonance spectroscopy imaging (MRSI) method, in a group of PHIV infected young adults (*N* = 28) compared to age and gender matched control sample (*N* = 28), and to find associations with HIV clinical factors and neurocognitive scores. MRSI data were acquired on a 3T scanner with a TE of 70 ms. Brain metabolites levels of total N-acetylaspartate (tNAA), total choline (tCho) and total creatine (tCre), as well as ratios of tNAA/tCre, tCho/tCre, and tNAA/tCho, were obtained from the whole brain level and evaluated at the level of gray matter (GM) and white matter (WM) tissue types and anatomical regions of interest (ROI). Our results indicate extensive metabolic abnormalities throughout the brains of PHIV infected subjects with significantly elevated levels of tCre and tCho, notably in GM regions. Decreases in tNAA and ratios of tNAA/tCre and tNAA/tCho were also found mostly in WM regions. These metabolic alterations indicate increased glial activation, inflammation, neuronal dysfunction, and energy metabolism in PHIV infected individuals, which correlated with a reduction in CD4 cell count, and lower cognitive scores. Our findings suggest that significant brain metabolite alterations and associated neurological complications persist in the brains of those with PHIV on long-term ART, and advocates the need for continued monitoring of their brain health.

## 1. Introduction

There were 38.4 million people living with HIV (PLWH) globally at the end of 2021, and an estimated 1.3 million women with HIV becoming pregnant each year (Joint United Nations Programme on HIV/AIDS [UNAIDS], 2022; World Health Organization [WHO], 2022). In the absence of intervention, the rate of mother-to-child perinatal HIV (PHIV) transmission during pregnancy, labor, delivery, or breastfeeding ranges from 20 to 25% (Irshad et al., 2022). Incidence of PHIV has remarkably subsided due to the success of antiretroviral therapy (ART), which has transformed HIV from a life-threatening disease to a manageable chronic infection. This has allowed HIV infected women to carry pregnancies with greatly reduced risk of PHIV transmission, and for children who acquired HIV perinatally to grow into adulthood with improved life expectancy and quality of life without major chronic comorbidities. However, significant neurological and neurocognitive complications persist in the brains of PHIV children and young adults despite the use of ART (Uban et al., 2015; Hoare et al., 2018; Sarma et al., 2019, 2021; Thakur et al., 2019; Van den Hof et al., 2019; Martin-Bejarano et al., 2021; Ruiz-Saez et al., 2021; Nichols, 2022). Extensive clinical studies in adult HIV patients indicated that most antiretrovirals either have poor penetration across the blood-brain barrier (BBB) or are effectively removed from the brain parenchyma, reducing their efficacy in suppressing HIV viral replication (Mzingwane and Tiemessen, 2017; Osborne et al., 2020). This may lead to the formation of viral reservoirs causing inflammation, astrocytic and neuronal dysregulation, and cell death (Nath, 2015; Ash et al., 2021). In pediatric brains, the evaluation of modern ART drug penetration has shown evidence of adequate viral suppression with suitable CNS concentrations of lamivudine, lopinavir, efavirenz, and nevirapine, but suboptimal concentrations of tenofovir, abacavir, and emtricitabine (Van den Hof et al., 2018). However, even under suppressive ART, the HIV long terminal repeat promoter remains active and produces viral particles and activates T-cells (Liu et al., 2020). Furthermore, the potential toxic effects to the brain from long term use of ART therapeutics is increasingly recognized (Margolis et al., 2014). While these complications have been extensively studied in adults with behaviorally acquired HIV, the long-term impact on the developing brains of PHIV children and young adults has not been characterized at the whole brain level. Additionally, many PHIV children who have survived into adulthood from earlier HIV therapeutic eras with potentially less effective and more toxic ART regimens may experience more severe brain injury (Margolis et al., 2014; Sarma et al., 2021). These impairments can also be attributed to environmental and socio-economic factors that disproportionately affect PHIV children (Nichols, 2022).

Most MRI-based neuroimaging studies on PHIV have focused on structural brain morphometry and microstructural alterations (Van den Hof et al., 2019; Martin-Bejarano et al., 2021; Nichols, 2022). Systematic reviews of brain imaging studies of PHIV mainly reported lower grey matter (GM) volumes and cortical thinning in PHIV subjects compared to healthy controls (Cohen et al., 2016; Lewis-de Los Angeles et al., 2017; Li J. et al., 2018; Nwosu et al., 2018). However, others found comparable (Sarma et al., 2014; Paul et al., 2018) or increased GM volumes (Sarma et al., 2014; Randall et al., 2017; Paul et al., 2018) in ART-treated PHIV patients. Some inconsistencies were found in the evaluation of white matter (WM) volumes (Sarma et al., 2014; Cohen et al., 2016; Randall et al., 2017; Li J. et al., 2018; Nwosu et al., 2018; Paul et al., 2018). Investigation of WM microstructure showed more concordance with lower fractional anisotropy (FA) and higher radial diffusivity (RD) in ART-treated PHIV compared to controls, as evidence of microstructural impairment of neurons and demyelination, respectively (Hoare et al., 2015; Li et al., 2015; Uban et al., 2015; Ackermann et al., 2016; Cohen et al., 2016; Jankiewicz et al., 2017). However, evaluation of regional mean- and axial- diffusivity values (MD, AD) were less conclusive.

By comparison, studies of brain metabolite concentrations in PHIV using proton magnetic resonance spectroscopy (MRS) were less common (Van den Hof et al., 2019). MRS allows for the acquisition of various metabolites, including N-acetylaspartate (NAA) as a proxy for neuronal density and viability, total choline (tCho) as an indicator of cell membrane turnover and gliosis, and total creatine (tCre) as a marker for cellular energy metabolism (Kirov et al., 2017). In general, contradictory findings have emerged from MRS studies of neurometabolites in PHIV (Keller et al., 2004; Nagarajan et al., 2012; Iqbal et al., 2016; Mbugua et al., 2016; Van Dalen et al., 2016; Robertson et al., 2018; Van den Hof et al., 2019; Graham et al., 2020). NAA concentrations were found to be higher for PHIV patients in the basal ganglia and frontal WM (Nagarajan et al., 2012; Mbugua et al., 2016), while other studies reported lower NAA in basal ganglia (Robertson et al., 2018) or no alterations (Keller et al., 2004). Similarly, findings on tCho in PHIV are not consistent, with either higher tCho reported in basal ganglia (Ashby et al., 2015; Mbugua et al., 2016), WM (Van Dalen et al., 2016), and frontal GM (Graham et al., 2020), or lower tCho in frontal WM (Keller et al., 2004), as well as several studies finding no significant differences compared to matched controls (Nagarajan et al., 2012; Iqbal et al., 2016). Levels of tCre were mostly lower for PHIV patients compared to healthy controls (Keller et al., 2004), and lower for PHIV infected children with ART after 12 weeks of age than those taking ART before 12 weeks (Mbugua et al., 2016). These discrepancies between researchers may be caused by differences in demographic and clinical characteristics such as the age of the subjects under investigation, medication history, stage of infection, co-morbidities, and co-infections (Keller et al., 2004; Holmes et al., 2017; Robertson et al., 2018), as well as differences in MRS processing and quantification methods which are affected by several factors such as SNR, baseline, or non-ideal experiment factors (Giapitzakis et al., 2019; Near et al., 2021; Marjanska et al., 2022).

Only a few of the aforementioned studies have included adult PHIV subjects (Nagarajan et al., 2012; Ashby et al., 2015; Iqbal et al., 2016), though not exclusively, for examining the long-term effects of HIV and ART on PHIV subjects that transitioned into adulthood. In addition, all these studies have used either a single-voxel MRS method, or a multi-voxel single-slice 2D-spectroscopic imaging (SI) method to evaluate metabolite changes in a few select brain anatomical regions (Van Dalen et al., 2016). By contrast, a volumetric proton magnetic resonance spectroscopic imaging (MRSI) method obtains data from multi-voxels in 3D to cover a subject’s entire head and thereby maps metabolite distributions from a large brain volume (Maudsley et al., 2009). To the best of our knowledge, no study investigating brain metabolites at the whole-brain level in PHIV has been published so far. Therefore, the goal of this cross-sectional study is to quantitate metabolite differences over a large brain volume, using a unique whole-brain MRSI method, between PHIV infected young adults compared to age and gender matched controls, and to associate brain metabolite levels with clinical laboratory data and neuropsychological (NP) scores. We hypothesized that the PHIV group would exhibit altered metabolite concentrations, and that the degree of those alterations would correlate with the severity of infection and cognitive impairments. The data and analysis presented herein are part of a larger multi-modal study designed to evaluate neurocognitive and neuroimaging outcomes including structural, diffusion, and metabolic MRI in a cohort of perinatally infected young adults. Results from the analysis of NP measures have been previously published (Willen et al., 2017).

## 2. Materials and methods

### 2.1. Participants

The Institutional Review Board of the University of Miami has approved this prospective and cross-sectional study; written informed consent was obtained from all participants. Inclusion criteria for all participants include 18 to 24 years of age and speaking English. Exclusion criteria for all participants include MRI contraindications, claustrophobia, a history of severe mental illness, brain injury/disease, and self-reported chronic alcohol or substance use. Perinatal HIV infection in PHIV subjects was confirmed through medical chart review. For the PHIV group, participants with a history of opportunistic infection or other neurological conditions (e.g., toxoplasmosis and stroke), and a documented IQ in the moderate to severe range of intellectual disability were also excluded in order to optimize the potential for identifying HIV-associated neurocognitive deficits. Controls were recruited to closely match the PHIV group by age and gender.

Twenty-nine PHIV and thirteen control subjects were enrolled in this study. Of these, one PHIV and three controls were unable to complete their MRI scans, leaving a final count of twenty-eight PHIV infected individuals (PHIV: *N* = 28) and ten controls. To closely match the sample size of the PHIV group, we selected 18 additional healthy control subjects from our other IRB-approved research studies that were performed at our facility, which recruited participants from the same source population as the PHIV study and used a similar MRI acquisition protocol, bringing the final number of controls to twenty-eight (Controls: *N* = 28). The additional control subjects were chosen following the same inclusion/exclusion criteria stated above.

### 2.2. MRSI acquisition and processing

MRI data were acquired using a 3T scanner (Siemens Trio) with multichannel phased-array detection. The protocol included a T_1_-weighted MPRAGE (TR/TE/TI = 2150/4.38/1100 ms, 1 × 1 × 1 mm^3^ resolution), a T_2_-weighted gradient echo, and a volumetric MRSI spin-echo EPSI sequence (TR/TE = 1710/70 ms, field of view: 280 × 280 × 180 mm^3^, slab thickness: 135 mm, nominal voxel volume: 0.31 cm^3^, Tacq = 26 min) that included an interleaved water reference acquisition obtained using a gradient-echo acquisition (TE = 6.3 ms).

**FIGURE 1:**
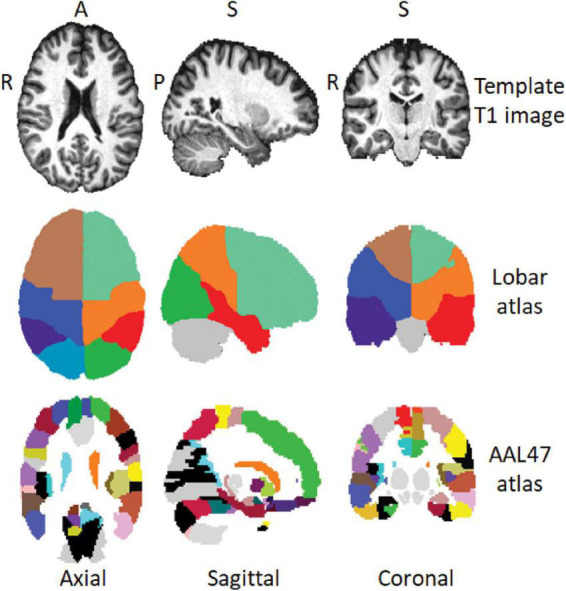
Axial, sagittal, and coronal view of a template T_1_-MRI in MNI space **(top row)**, regions of interest (ROI) delineations in the hemispheric lobar **(middle row)** and AAL47 atlases **(bottom row)**. A, anterior; P, posterior; S, superior; R, right.

Magnetic resonance spectroscopy imaging (MRSI) data were processed using our MIDAS software (details in Maudsley et al., 2006, 2009) to obtain signal-normalized and cerebrospinal fluid (CSF) partial volume corrected metabolite concentration maps in institutional units for total NAA (tNAA), which includes contributions from NAA and N-Acetylaspartylglutamate (NAAG), tCre, and tCho. Briefly, the processing and analysis steps in MIDAS include spectral frequency drift and phase shift corrections, residual water signal filtering, baseline and spectral fits, incorporation of tissue and CSF partial volumes, and spectral quality criteria (linewidth, CSF partial volume, outlier removal). For each individual subject, the gradient echo water signal reference data was used for the metabolite signal normalization and the segmentation of T_1_ images was used to obtain GM, WM, and CSF partial volume maps, which were then were spatially registered to a template T_1_-MRI in MNI space. The metabolite images were reconstructed to 64 × 64 × 32 points with the nominal voxel volume of 0.313 cc that was increased to approximately 1.5 cc following spatial smoothing. After reconstructing individual subject tNAA, tCre, and tCho maps, metabolite concentrations and metabolite ratios of tCho/tCre, tNAA/tCre, and tNAA/tCho were evaluated at specific regions of interest (ROI) using brain atlases in MNI space. We used the 9-region hemispheric lobar atlas (Maudsley et al., 2006) which identifies the frontal, parietal, temporal, and occipital lobes (from the left and right brain) with the cerebellum, and the AAL47 atlas (Tzourio-Mazoyer et al., 2002) which identifies 47 cortical and deep GM ROIs (Figure 1). Data quality was controlled by including only spectra from voxels with < 30% CSF and fitted spectral linewidths of ≤ 12 Hz. For the lobar atlas, metabolite concentration corresponding to 70% of GM/WM in each of the 8 hemispheric lobes was calculated by regressing the tissue partial volume against the metabolite concentration and extrapolating it to 100% of GM or WM. The AAL47 atlas also contains delineation of the cerebellum and the data from which was not used as it is already defined in the lobar atlas, reducing the number of analyzed ROIs to 46 (List of all AAL47 ROIs with corresponding abbreviations is provided in Supplementary Table 1). Three ROIs in the AAL47 atlas are also labeled as frontal, occipital, and temporal gyri. These do not correspond to the entire frontal, occipital, and temporal lobes, as defined in the lobar atlas, but are sub-regions of smaller sizes and therefore were included in the analysis.

### 2.3. Clinical and neuropsychological data

HIV-associated clinical factors were collected from PHIV subjects including CD4, nadir CD4 (CD4ndr), viral load (VL), and medication regimen taken at time of study. The study protocol also included NP assessment battery designed to capture the participant’s performance in overall cognition as well as in specific domains of attention, working memory, immediate and delayed recall, fine motor speed, and cognitive flexibility. Tests administered included: Full Scale IQ (FSIQ) index from the Wechsler Abbreviate Scale of Intelligence (Wechsler, 1999); Digit Span (DS) and Letter Number Sequencing (LNS) from the Wechsler Adult Intelligence Scale, Third Edition (Wechsler, 1997); Immediate Memory Index (IMI), Delayed Memory Index (DMI) and Symbol Span (SS) from the Wechsler Memory Scale, Fourth Edition (Wechsler, 2009); Trail Making Test, Condition 4 (TRLS), and the Verbal Fluency Test by Letter (LET) and category (CAT) from the Delis Kaplan Executive Function System (Delis et al., 2001).

### 2.4. Analyses and statistical methods

Demographic group differences were analyzed using Fisher’s Exact test for categorical variables and Chi-Squared test for continuous data. Neuroimaging outcomes were analyzed at two levels: at the lobar level to examine group differences in specific tissue types (GM, WM), and at the level of brain anatomical ROIs identified by the AAL47 atlas. All MRSI measures were assessed for normality and homogeneity of variances using Shapiro-Wilk and Levene’s tests, respectively. For each measure, outliers with more than three standard deviations from the mean were removed. Using both lobar and AAL47 atlases, we first compared average metabolite concentrations/ratios between the left and right brain hemispheres using paired *t*-tests, for controls and PHIV subjects separately, to determine if corresponding left/right ROIs can be combined for the subsequent analyses. We found significant asymmetry of metabolite distribution between the left and right brain, particularly in the PHIV group, and as such corresponding left/right ROIs would not be combined.

We then performed between-group comparisons of metabolite concentrations and ratios in each brain lobe, at the level of GM and WM tissue types, using analysis of covariance (ANCOVA), or non-parametric equivalent (Quade, 1967) when normality and homogeneity assumptions were not met, controlling for age and gender as covariates. MRSI measures were then correlated with clinical variables (CD4, CD4ndr, and VL) and NP scores (FSIQ, DS, LNS, IMI, DMI, SS, TRLS, LET, CAT). We repeated the between-group ANCOVA analysis at the brain anatomical ROI level using MRSI measures evaluated at regions defined by the AAL47 atlas. Finally, we performed correlation analyses with clinical and NP variables. All statistical tests were performed using R programming language, with significance at *p* < 0.05 corrected for multiple comparisons by false discovery rate (FDR) using the Benjamini-Hochberg procedure (Benjamini and Hochberg, 1995).

## 3. Results

### 3.1. Demographic characteristics

Table 1 summarizes the demographic information of participants included in this study. There were no significant differences with respect to gender and age; however, there was a difference between the two groups’ ethnicity, with PHIV subjects being predominantly African-American. This is due to the addition of the eighteen control subjects from other studies. While these additions were matched for age and gender in the PHIV group, we could not match them on ethnicity. Other factors such as education level and NP data were not collected for these additional control subjects as neurocognitive assessment was not part of the design of those studies in which they were enrolled. At the time of evaluation, a majority of PHIV participants (78.6%) were taking ART therapeutics. However, clinical outcomes show that only a few (32.2%) were virally suppressed [i.e., a viral load (VL) of < 200 copies per mL of blood] with high VL and sub-optimal CD4 cell count found in several participants. This may suggest poor adherence to ART among them.

**TABLE 1:** Participant characteristics.

Participant characteristics	PHIV (*N* = 28)	Control (*N* = 28)	*p*-value
Gender			0.999
Male	14	13	
Female	14	15	
Age	20.3 ± 1.9	20.8 ± 1.7	0.608
Ethnicity			0.016*
Asian	0 (0%)	3 (10.7%)	
African-American	23 (82.1%)	11 (39.3%)	
White (non-hispanic)	1 (3.6%)	3 (10.7%)	
Hispanic white	2 (7.1%)	5 (17.9%)	
Hispanic other	2 (7.1%)	6 (21.4%)	
Education			
< High school	12 (42.8%)	−	
High school or GED	8 (28.6%)	−	
Some college	8 (28.6%)	−	
Medical variables			
CD4 (copies/mm^3^)	402.1 ± 291.3	−	
Nadir CD4 (copies/mm^3^)	168.6 ± 162	−	
Viral load (log_10_ copies/mL)	2.98 ± 1.02	−	
Virally suppressed	9 (32.2%)	−	
On ART/Other medication	22 (78.6%)	−	
History of meningitis	1 (3.6%)	−	
History of encephalopathy	8 (28.6%)	−	

*p < 0.05.

Eight participants had a history of HIV associated encephalopathy and one patient had a history of unspecified meningitis, with the remaining nineteen subjects having no history of neurological injuries. These nine subjects were initially analyzed as a separate group from the remaining PHIV subjects to determine if incidence of encephalopathy/meningitis had any effect on MRSI measures. There were no significant differences on any of the dependent variables between PHIV participants with or without encephalopathy/meningitis (data not shown) and all PHIV participants were subsequently analyzed as a single group. The same analysis was performed on NP measures in our published study, and similarly it was determined that history of encephalopathy/meningitis had no effect on cognitive outcomes (Willen et al., 2017).

### 3.2. Brain lobar level analysis by tissue types

**FIGURE 2:**
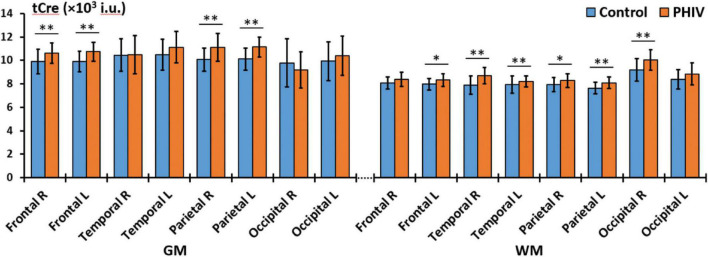
Comparison of mean total creatine (tCre) values in institutional units (i.u.) between the control (blue) and perinatally acquired HIV (PHIV) (orange) groups in the GM and WM of all 8 brain hemispheric lobar regions. Bars indicate standard deviation. Asterisks indicate significant difference [*p < 0.05, ^**^p < 0.01; false discovery rate (FDR) corrected]. R, right; L, left; GM, gray matter; WM, white matter.

**FIGURE 3:**
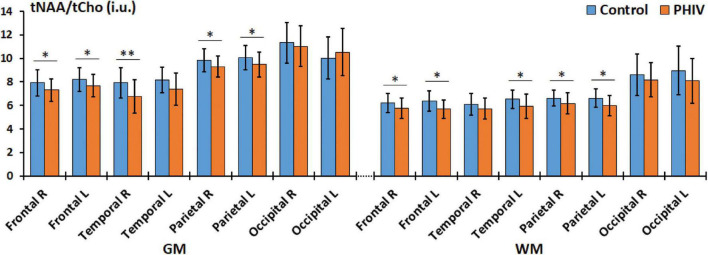
Comparison of mean total N-acetylaspartate (tNAA)/total choline (tCho) values in institutional units (i.u.) between the control (blue) and perinatally acquired HIV (PHIV) (orange) groups in the GM and WM of all 8 brain hemispheric lobar regions. Bars indicate standard deviation. Asterisks indicate significant difference [*p < 0.05, ^**^p < 0.01; false discovery rate (FDR) corrected]. R, right; L, left; GM, gray matter; WM, white matter.

Between-group comparisons from the hemispheric lobar level analysis of metabolite concentrations and ratios are presented in Tables 2, 3, respectively. Results showed that PHIV subject had significantly higher tNAA only in the GM of the left parietal lobe compared to controls. Concentrations of tCre were significantly higher for the PHIV group in all the GM of both left and right, frontal and occipital lobes (Figure 2). Elevated levels of tCre were also found in the WM of all lobes, except the right frontal and left occipital lobes. Similarly, concentrations of tCho in were higher for the PHIV group in the GM and WM of nearly all the lobes except in the occipital lobe. Ratios of tNAA/tCre were lower for PHIV in all the WM lobes, and in most of the GM except for the parietal and the left occipital lobes. We also see lower tNAA/tCho in PHIV in both the GM and WM of all lobes except for the occipital lobe (Figure 3). Only ratios tCho/tCre were not significantly different in any lobes. No significant differences were found in the cerebellum.

**TABLE 2:** Analysis of covariance (ANCOVA) results showing group mean, standard deviation (sd), and *p*-values for metabolite concentrations of total N-acetylaspartate (tNAA), total creatine (tCre), and total choline (tCho) in the brain hemispheric lobar regions.

ROI (lobar atlas)		tNAA			tCre			tCho		
		**Control mean (sd)^#^**	**PHIV mean (sd)^#^**	***p***	**Control mean (sd)^#^**	**PHIV mean (sd)^#^**	***p***	**Control mean (sd)^#^**	**PHIV mean (sd)^#^**	***p***
GM	Frontal R	14.37 (1.31)	14.68 (0.85)	0.685	9.9 (1.05)	10.61 (0.87)	0.009*	1.95 (0.22)	2.1 (0.19)	0.016*
	Frontal L	14.56 (0.87)	15.11 (1.03)	0.149	9.92 (0.87)	10.74 (0.8)	0.003*	1.97 (0.24)	2.17 (0.23)	0.007*
	Temporal R	14.69 (1.65)	13.99 (1.98)	0.342	10.44 (1.39)	10.47 (1.62)	0.881	1.78 (0.37)	1.91 (0.36)	0.281
	Temporal L	14.64 (1.42)	14.62 (1.91)	0.99	10.49 (1.33)	11.13 (1.35)	0.079	1.85 (0.31)	2.07 (0.31)	0.03*
	Parietal R	15.08 (0.88)	15.78 (1.05)	0.098	10.06 (0.97)	11.1 (1.17)	0.003*	1.36 (0.2)	1.55 (0.21)	0.007*
	Parietal L	14.62 (1.06)	15.83 (1.44)	0.005*	10.11 (0.94)	11.13 (0.69)	0.003*	1.4 (0.19)	1.64 (0.33)	<0.001*
	Occipital R	13.56 (2.05)	13.35 (2.08)	0.685	9.77 (2.06)	9.18 (1.54)	0.397	1.21 (0.29)	1.12 (0.31)	0.281
	Occipital L	13.66 (1.82)	14.11 (1.97)	0.709	9.93 (1.64)	10.38 (1.66)	0.415	1.45 (0.33)	1.41 (0.37)	0.626
WM	Frontal R	14.16 (0.94)	13.59 (1.15)	0.11	8.06 (0.52)	8.37 (0.59)	0.076	2.39 (0.17)	2.5 (0.28)	0.044*
	Frontal L	14.08 (0.82)	13.61 (0.99)	0.11	7.98 (0.49)	8.32 (0.53)	0.036*	2.31 (0.18)	2.46 (0.25)	0.016*
	Temporal R	14.04 (1.49)	14.09 (1.44)	0.884	7.89 (0.77)	8.69 (0.69)	0.003*	2.43 (0.29)	2.7 (0.33)	0.007*
	Temporal L	14.21 (1.12)	13.7 (1.05)	0.11	7.94 (0.75)	8.22 (0.47)	0.009*	2.3 (0.23)	2.44 (0.22)	0.023*
	Parietal R	14.59 (0.95)	14.02 (1.03)	0.098	7.95 (0.61)	8.29 (0.58)	0.034*	2.29 (0.21)	2.41 (0.21)	0.03*
	Parietal L	14.15 (0.82)	13.93 (1.91)	0.342	7.62 (0.49)	8.08 (1.35)	0.008*	2.2 (0.21)	2.36 (0.31)	0.007*
	Occipital R	16.2 (1.22)	15.98 (1.39)	0.676	9.19 (0.97)	10.01 (0.87)	0.009*	1.98 (0.3)	2.09 (0.26)	0.222
	Occipital L	15.03 (1.55)	14.87 (1.39)	0.846	8.38 (0.84)	8.84 (0.94)	0.097	1.78 (0.21)	1.87 (0.31)	0.222
Cerebellum		14.13 (0.96)	13.4 (1.22)	0.056	13.63 (1.61)	13.6 (1.44)	0.902	3.13 (0.36)	3.06 (0.31)	0.594

^#^Values are (× 10^3^) expressed in institutional units (i.u). *p < 0.05 (FDR corrected). R, right; L, left; GM, gray matter; WM, white matter.

**TABLE 3:** Analysis of covariance (ANCOVA) results showing group mean, standard deviation (sd), and *p*-values for metabolite ratios of total N-acetylaspartate (tNAA)/total creatine (tCre), total choline (tCho)/tCre, and tNAA/tCho in the brain hemispheric lobar regions.

ROI (lobar atlas)		tNAA/tCre			tCho/tCre			tNAA/tCho		
		**Control mean (sd)^#^**	**PHIV mean (sd)^#^**	***p***	**Control mean (sd)^#^**	**PHIV mean (sd)^#^**	***p***	**Control mean (sd)^#^**	**PHIV mean (sd)^#^**	***p***
GM	Frontal R	1.43 (0.1)	1.34 (0.11)	<0.001*	0.19 (0.02)	0.18 (0.02)	0.956	7.92 (1.13)	7.3 (0.94)	0.024*
	Frontal L	1.49 (0.13)	1.39 (0.13)	0.005*	0.2 (0.03)	0.19 (0.03)	0.956	8.21 (1.01)	7.66 (0.95)	0.024*
	Temporal R	1.25 (0.14)	1.12 (0.17)	0.005*	0.14 (0.03)	0.14 (0.03)	0.956	7.92 (1.28)	6.77 (1.43)	0.005*
	Temporal L	1.35 (0.17)	1.25 (0.18)	0.026*	0.16 (0.02)	0.17 (0.02)	0.956	8.18 (1.08)	7.39 (1.36)	0.04
	Parietal R	1.36 (0.12)	1.32 (0.1)	0.089	0.11 (0.02)	0.11 (0.02)	0.956	9.86 (0.98)	9.31 (0.9)	0.031*
	Parietal L	1.4 (0.12)	1.38 (0.21)	0.232	0.13 (0.02)	0.13 (0.05)	0.956	10.04 (1.04)	9.49 (0.9)	0.033*
	Occipital R	1.37 (0.15)	1.3 (0.15)	0.033*	0.11 (0.03)	0.11 (0.02)	0.956	11.33 (1.74)	11.04 (1.72)	0.52
	Occipital L	1.35 (0.21)	1.34 (0.16)	0.713	0.13 (0.03)	0.13 (0.02)	0.956	10.02 (1.77)	10.53 (2.02)	0.426
WM	Frontal R	1.8 (0.15)	1.68 (0.14)	0.005*	0.3 (0.03)	0.3 (0.03)	0.956	6.21 (0.79)	5.76 (0.86)	0.032*
	Frontal L	1.82 (0.15)	1.68 (0.15)	0.005*	0.3 (0.03)	0.3 (0.03)	0.956	6.38 (0.89)	5.68 (0.76)	0.013*
	Temporal R	1.86 (0.18)	1.74 (0.21)	0.021*	0.32 (0.04)	0.33 (0.05)	0.956	6.1 (0.9)	5.71 (0.9)	0.055
	Temporal L	1.85 (0.16)	1.72 (0.17)	0.005*	0.3 (0.03)	0.3 (0.03)	0.956	6.52 (0.8)	5.93 (1.05)	0.024*
	Parietal R	1.88 (0.15)	1.75 (0.16)	0.005*	0.29 (0.03)	0.3 (0.03)	0.956	6.62 (0.69)	6.17 (0.91)	0.033*
	Parietal L	1.89 (0.15)	1.76 (0.18)	0.005*	0.29 (0.03)	0.29 (0.02)	0.956	6.6 (0.79)	5.99 (1.36)	0.012*
	Occipital R	1.83 (0.18)	1.69 (0.18)	0.011*	0.22 (0.03)	0.22 (0.03)	0.956	8.6 (1.76)	8.18 (1.47)	0.392
	Occipital L	1.84 (0.13)	1.7 (0.16)	0.005*	0.22 (0.03)	0.22 (0.03)	0.648	8.97 (2.06)	8.08 (1.88)	0.13
Cerebellum		1.03 (0.09)	0.97 (0.1)	0.056	0.23 (0.02)	0.22 (0.02)	0.056	4.68 (0.72)	4.4 (0.53)	0.205

^#^Values are expressed in institutional units (i.u). *p < 0.05 (FDR corrected). R, right; L, left; GM, gray matter; WM, white matter.

**FIGURE 4:**
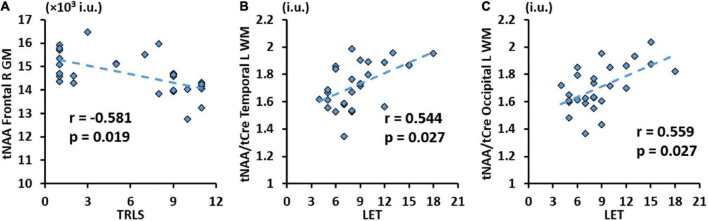
Significant correlation between trail making test (TRLS) and total N-acetylaspartate (tNAA) in the GM of the right frontal gyrus **(A)**, and between Letter Fluency Test (LET) and total N-acetylaspartate (tNAA)/total choline (tCho) in WM of the left temporal **(B)** and occipital **(C)** gyri with respective *r* and *p*-values [false discovery rate (FDR) corrected].

Correlation analysis results between metabolic concentrations and ratios derived from the lobar atlas and clinical variables are summarized in Table 4 showing only the metrics where significant correlations were found. Results show that tCre was negatively correlated in 3 GM regions (frontal and parietal lobes) with CD4 cell count, while tNAA/tCre was positively correlated in the WM of all 8 hemispheric brain lobes with CD4 cell count. We also see positive correlation between tCho/tCre in the right parietal GM and nadir CD4. No significant correlations were found with respect to VL. Correlation analysis with NP variables only showed a negative correlation between tNAA in the right frontal GM and TRLS (Figure 4A), and positive correlations between tNAA/tCre in the left temporal and occipital WM and LET (Figures 4B, C).

**TABLE 4:** Pearson correlation results between metabolite concentrations and ratios from the lobar atlas and clinical variables (CD4, CD4 nadir, and VL), showing r and *p*-values for all metrics with significant correlations.

Metric	ROI (lobar atlas)	CD4		CD4 nadir		VL (log_10_)	
		***r***	***p***	***r***	***p***	***r***	***p***
tCre	Frontal R GM	-0.525	0.049*	-0.226	0.474	0.199	0.895
tCre	Parietal R GM	-0.499	0.049*	-0.18	0.536	0.127	0.895
tCre	Parietal L GM	-0.491	0.049*	-0.258	0.463	0.002	0.992
tNAA/tCre	Frontal R WM	0.473	0.034*	0.387	0.4	-0.135	0.723
tNAA/tCre	Frontal L WM	0.524	0.034*	0.413	0.4	-0.123	0.723
tNAA/tCre	Temporal R WM	0.582	0.019*	0.233	0.446	-0.222	0.662
tNAA/tCre	Temporal L WM	0.492	0.034*	0.25	0.446	-0.276	0.662
tNAA/tCre	Parietal R WM	0.459	0.034*	0.224	0.446	-0.068	0.843
tNAA/tCre	Parietal L WM	0.454	0.034*	0.278	0.446	-0.205	0.662
tNAA/tCre	Occipital R WM	0.505	0.034*	0.102	0.825	-0.049	0.861
tNAA/tCre	Occipital L WM	0.475	0.034*	0.068	0.858	0.028	0.891
tCho/tCre	Parietal R GM	0.452	0.232	0.592	0.018*	-0.536	0.053

*p < 0.05 (FDR corrected). R, right; L, left.

### 3.3. Group comparisons by anatomical ROIs

**FIGURE 5:**
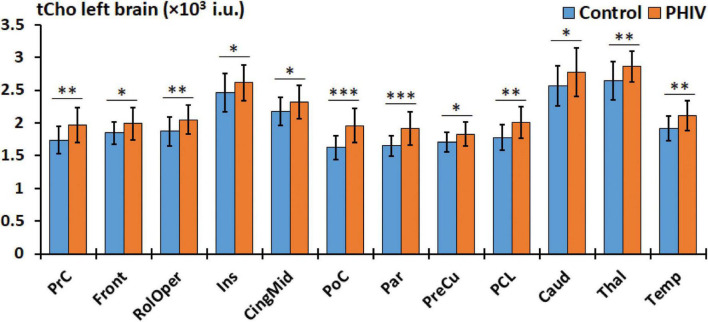
Comparison of mean total choline (tCho) values in institutional units (i.u.) between the control (blue) and perinatally acquired HIV (PHIV) (orange) groups in AAL47 regions of interests (ROIs) from the left brain. Bars indicate standard deviation. Only ROIs with significant differences are shown [*p < 0.05, ^**^p < 0.01, ^***^p < 0.001; false discovery rate (FDR) corrected].

**FIGURE 6:**
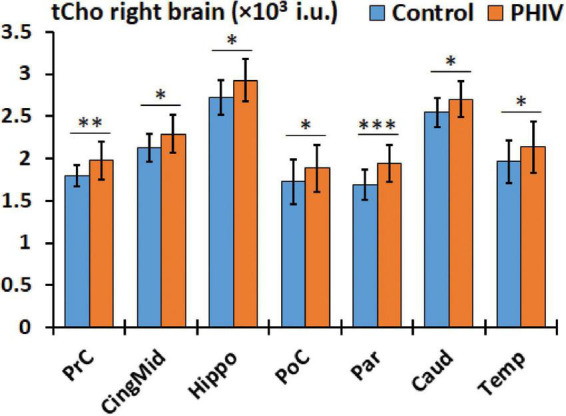
Comparison of mean total choline (tCho) values in institutional units (i.u.) between the control (blue) and perinatally acquired HIV (PHIV) (orange) groups in AAL47 regions of interests (ROIs) from the right brain. Bars indicate standard deviation. Only ROIs with significant differences are shown [*p < 0.05, ^**^p < 0.01, ^***^p < 0.001; false discovery rate (FDR) corrected].

**FIGURE 7:**
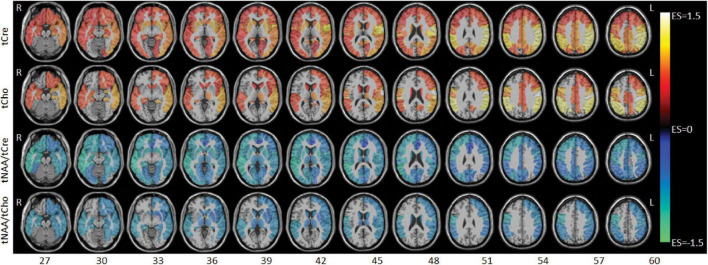
Axial slices showing between-group effect size (ES) maps for total creatine (tCre), total choline (tCho), total N-acetylaspartate (tNAA)/tCre, and tNAA/tCho (top to bottom). ES values range from –1.5 to 1.5 with negative values (cool colors) denoting lower metabolite levels for the perinatally acquired HIV (PHIV) groups, and positive values (warm colors) denoting higher metabolite levels for the PHIV + group. Abbreviations denote the right (R) and left (L) side of the brain.

Between-group comparison results of tNAA, tCre, and tCho evaluated in the AAL47 atlas are summarized in Tables 5, 6 for right-brain and left-brain ROIs, respectively, showing only the ROIs where significant differences are found. Significantly elevated concentrations of tCre were found in the PHIV group compared to controls, primarily in the left brain with (18 ROIs) as opposed to the right brain (13 ROIs). Similarly, tCho showed some lateralization in the left brain with elevated concentrations for PHIV subjects at 12 ROIs compared to 7 ROIs from the right brain as seen in Figures 5, 6, respectively (only ROIs with significant *p*-values were plotted). This pattern could also be observed for ratios of tNAA/tCho with significant decreases for PHIV subjects in 14 left-brain ROIs compared to 8 right-brain ROIs. Only ratios of tNAA/tCre exhibited symmetry with near equal number of left (19 ROIs) and right-brain (20 ROIs) regions showing significant decreases for the PHIV group. Figure 7 maps these metabolic alterations by visualizing between-group effect sizes (ES), calculated by Cohen’s D, for tCre, tCho, tNAA/tCre, and tNAA/tCho at each ROI where significant differences are found, tNAA and tCho/tCre were not represented as they had no significant changes. Positive ES signifies elevated levels for PHIV whereas negative ES show reduced metabolite levels. The complete results from all AAL47 ROIs for metabolites and ratios are provided in Supplementary Tables 2, 3, respectively.

**TABLE 5:** Analysis of covariance (ANCOVA) results showing group mean, standard deviation (sd), and *p*-values for metabolite concentrations of total N-acetylaspartate (tNAA), total creatine (tCre), and total choline (tCho) in right brain regions of interests (ROIs) of the AAL47 atlas.

ROI (AAL47 atlas right brain)	tNAA			tCre			tCho		
	**Control mean (sd)^#^**	**PHIV mean (sd)^#^**	***p***	**Control mean (sd)^#^**	**PHIV mean (sd)^#^**	***p***	**Control mean (sd)^#^**	**PHIV mean (sd)^#^**	***p***
PrC	14.48 (1.21)	14.71 (1.41)	0.925	8.75 (0.82)	9.35 (0.72)	0.024*	1.8 (0.13)	1.98 (0.22)	0.003*
Front	13.65 (1.21)	13.28 (1.17)	0.695	8.46 (0.78)	8.82 (0.73)	0.048*	1.92 (0.17)	2.02 (0.22)	0.091
RolOper	14.6 (0.88)	14.56 (0.8)	0.925	9.07 (0.71)	9.61 (0.79)	0.022*	1.97 (0.21)	2.05 (0.21)	0.13
Ins	15.34 (0.98)	15.37 (0.86)	1 10.06 (0.83)		10.68 (0.85)	0.013*	2.54 (0.27)	2.64 (0.27)	0.15
CingMid	14.94 (0.8)	15.12 (0.92)	0.849	9.6 (0.64)	10.07 (0.65)	0.028*	2.13 (0.21)	2.29 (0.26)	0.021*
Hippo	13.48 (1.27)	13.28 (0.98)	0.695	9.22 (1.18)	9.84 (0.65)	0.022*	2.72 (0.26)	2.93 (0.28)	0.024*
Occ	14.72 (1.24)	14.6 (0.83)	0.765	8.77 (0.85)	9.34 (0.91)	0.047*	1.52 (0.21)	1.58 (0.22)	0.451
PoC	14.55 (0.93)	14.5 (1.04)	0.799	9.02 (0.79)	9.53 (0.75)	0.041*	1.73 (0.18)	1.89 (0.22)	0.019*
Par	14.65 (0.9)	15.3 (0.97)	0.219	9.07 (0.8)	9.93 (0.84)	0.004*	1.69 (0.18)	1.94 (0.21)	<0.001*
PreCu	15.02 (0.74)	15.11 (0.74)	1 9.59 (0.73)		10.23 (0.86)	0.025*	1.7 (0.15)	1.78 (0.18)	0.094
Caud	14.05 (1.17)	13.76 (1.03)	0.695	8.89 (0.76)	9.62 (0.93)	0.011*	2.55 (0.25)	2.7 (0.31)	0.036*
Put	14.33 (1.15)	14.15 (1.11)	0.799	9.69 (0.83)	10.38 (0.69)	0.01*	2.34 (0.25)	2.49 (0.26)	0.053
Temp	14.6 (1.02)	14.33 (1.13)	0.695	9.22 (0.81)	9.79 (1.12)	0.044*	1.97 (0.25)	2.14 (0.27)	0.034*

^#^Values are (× 10^3^) expressed in institutional units (i.u). *p < 0.05 (FDR corrected).

**TABLE 6:** Analysis of covariance (ANCOVA) results showing group mean, standard deviation (sd), and *p*-values for metabolite concentrations of total N-acetylaspartate (tNAA), total creatine (tCre), and total choline (tCho) in left brain regions of interests (ROIs) of the AAL47 atlas.

ROI (AAL47 atlas left brain)	tNAA			tCre			tCho		
	**Control mean (sd)^#^**	**PHIV mean (sd)^#^**	***p***	**Control mean (sd)^#^**	**PHIV mean (sd)^#^**	***p***	**Control mean (sd)^#^**	**PHIV mean (sd)^#^**	***p***
PrC	14.43 (1.23)	14.95 (1.37)	0.695	8.71 (0.88)	9.52 (0.72)	0.01*	1.74 (0.21)	1.97 (0.27)	0.005*
Front	13.91 (0.73)	13.79 (1)	0.799	8.55 (0.66)	9 (0.73)	0.03*	1.85 (0.17)	1.99 (0.25)	0.033*
RolOper	14.36 (0.72)	14.59 (0.89)	0.799	8.97 (0.72)	9.69 (0.57)	0.003*	1.87 (0.22)	2.05 (0.22)	0.006*
SMA	13.97 (1.14)	13.93 (1.1)	0.849	9.08 (0.99)	9.74 (1.05)	0.042*	1.99 (0.23)	2.13 (0.24)	0.072
Ins	15.03 (0.77)	15.17 (0.99)	0.853	9.95 (0.81)	10.62 (0.8)	0.01*	2.46 (0.29)	2.62 (0.27)	0.036*
CingAnt	14.56 (1.26)	14.63 (1.15)	0.987	9.67 (1.15)	10.38 (0.93)	0.025*	2.76 (0.39)	2.89 (0.42)	0.192
CingMid	15.09 (0.85)	15.42 (0.93)	0.695	9.67 (0.63)	10.27 (0.69)	0.01*	2.18 (0.22)	2.32 (0.25)	0.038*
Hippo	13.51 (1.13)	13.34 (1.18)	0.765	9.18 (1.05)	9.77 (0.73)	0.031*	2.75 (0.24)	2.88 (0.23)	0.053
Calc	15.17 (1.07)	14.98 (1.05)	0.765	9.18 (1)	9.57 (0.85)	0.038*	1.61 (0.2)	1.6 (0.22)	0.854
Cu	14.35 (1.03)	14.58 (1.23)	0.849	8.79 (0.82)	9.4 (0.91)	0.028*	1.46 (0.21)	1.47 (0.24)	0.942
Ling	14.93 (1.04)	14.74 (0.94)	0.695	10.15 (1.43)	10.77 (1.24)	0.031*	1.85 (0.28)	1.88 (0.29)	0.373
Occ	13.73 (1.08)	14.24 (1.13)	0.511	8.47 (0.75)	9.21 (0.87)	0.01*	1.53 (0.21)	1.6 (0.26)	0.428
PoC	13.9 (0.97)	14.64 (1.2)	0.222	8.76 (0.69)	9.65 (0.67)	0.001*	1.62 (0.18)	1.96 (0.26)	<0.001*
Par	14.16 (0.96)	15.02 (1.24)	0.207	8.92 (0.71)	9.84 (0.73)	0.001*	1.65 (0.16)	1.91 (0.26)	<0.001*
PreCu	14.79 (0.69)	15.02 (0.87)	0.799	9.27 (0.73)	9.9 (0.76)	0.01*	1.71 (0.15)	1.83 (0.19)	0.021*
PCL	13.66 (1.24)	14.4 (1.32)	0.375	9.32 (0.95)	10.14 (1.27)	0.028*	1.78 (0.19)	2 (0.24)	0.002*
Caud	14.46 (1.14)	14.04 (1.03)	0.436	9.03 (0.96)	9.71 (0.93)	0.025*	2.57 (0.31)	2.78 (0.37)	0.027*
Put	14.39 (0.93)	14.17 (0.93)	0.695	9.61 (0.89)	10.45 (0.79)	0.008*	2.4 (0.23)	2.53 (0.23)	0.051
Thal	14.72 (1.35)	14.83 (1.4)	1 9.32 (0.96)		9.84 (0.79)	0.051	2.65 (0.29)	2.86 (0.23)	0.006*
Temp	14.62 (0.99)	14.53 (1.11)	0.911	9.26 (0.71)	9.93 (0.93)	0.01*	1.92 (0.19)	2.11 (0.23)	0.006*

^#^Values are (× 10^3^) expressed in institutional units (i.u). *p < 0.05 (FDR corrected).

**FIGURE 8:**
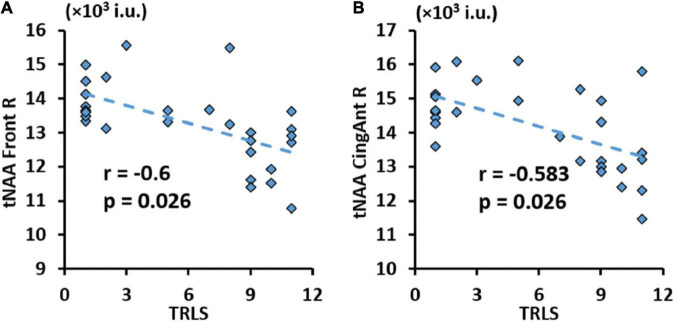
Significant negative correlation between trail making test (TRLS) and total N-acetylaspartate (tNAA) in the right frontal gyrus **(A)**, and right anterior cingulum **(B)** with respective *r* and *p*-values [false discovery rate (FDR) corrected].

Finally, results from correlations between metabolic concentrations/ratios derived from the AAL47 atlas and clinical variables are summarized in Table 7, showing only metrics with significant correlations. Similar to lobar results, tCre was negatively correlated and tNAA/tCre was positively correlated with CD4 cell count, respectively, at multiple ROIs. We also observed negative association between tNAA in the left hippocampus and VL. No significant correlations were found with respect to CD4 nadir. Of the NP variables, only TRLS showed significant negative correlation with tNAA in the right frontal gyrus (Figure 8A) and right anterior cingulum (Figure 8B).

**TABLE 7:** Pearson correlation results between metabolite concentrations and ratios from the AAL47 atlas and clinical variables (CD4, CD4 nadir, and VL), showing *r* and *p*-values for all metrics with significant correlations.

Metric	ROI (AAL47 atlas)	CD4		CD4 nadir		VL (log_10_)	
		***r***	***p***	***r***	***p***	***r***	***p***
NAA	Hippo L	0.202	0.884	0.146	0.99	-0.587	0.048*
tCre	Front L	-0.593	0.024*	-0.187	0.501	0.193	0.989
tCre	RolOper R	-0.514	0.036*	-0.207	0.501	0.028	0.994
tCre	CingMid R	-0.521	0.036*	-0.385	0.271	0.131	0.989
tCre	Hippo R	-0.52	0.036*	-0.365	0.285	0.063	0.989
tCre	Calc R	-0.545	0.036*	-0.233	0.501	0.103	0.989
tCre	Occ L	-0.508	0.036*	-0.221	0.501	0.08	0.989
tCre	Occ R	-0.588	0.024*	-0.084	0.739	0.166	0.989
tCre	Temp R	-0.534	0.036*	-0.405	0.27	0.171	0.989
tNAA/tCre	PrC L	0.497	0.043*	0.469	0.3	-0.208	0.515
tNAA/tCre	RolOper L	0.476	0.043*	0.333	0.329	-0.34	0.309
tNAA/tCre	RolOper R	0.638	0.006*	0.289	0.376	-0.299	0.364
tNAA/tCre	SMA L	0.443	0.048*	0.315	0.329	-0.036	0.932
tNAA/tCre	SMA R	0.506	0.042*	0.231	0.425	-0.196	0.515
tNAA/tCre	Ins R	0.46	0.045*	0.307	0.329	-0.386	0.309
tNAA/tCre	Hippo L	0.699	0.002*	0.369	0.3	-0.275	0.364
tNAA/tCre	Hippo R	0.522	0.042*	0.139	0.566	-0.365	0.309
tNAA/tCre	Occ L	0.504	0.042*	0.519	0.262	-0.409	0.309
tNAA/tCre	Fu L	0.482	0.043*	0.203	0.501	-0.048	0.91
tNAA/tCre	Fu R	0.514	0.042*	0.168	0.532	-0.325	0.329
tNAA/tCre	PoC L	0.448	0.047*	0.407	0.3	-0.267	0.364
tNAA/tCre	PoC R	0.457	0.045*	0.25	0.425	-0.336	0.309
tNAA/tCre	PreCu L	0.483	0.043*	0.311	0.329	-0.291	0.364
tNAA/tCre	PCL L	0.568	0.025*	0.08	0.739	-0.131	0.7
tNAA/tCre	Caud R	0.47	0.043*	0.173	0.531	-0.361	0.309
tNAA/tCre	GP R	0.451	0.047*	0.246	0.425	0.007	0.972
tNAA/tCre	Cereb L	0.469	0.043*	0.162	0.532	0.032	0.932

*p < 0.05 (FDR corrected). Abbreviations for hemispheric regions: R, right; L, left.

## 4. Discussion

The results of this study show that significant alterations of proton MRSI observed metabolites occur in the whole brain of adults perinatally infected with HIV, who are taking ART, compared to healthy controls sourced from the same local population. Using the data acquired with our unique MRSI acquisition technique, these metabolite alterations can be quantified and mapped at the whole-brain level using appropriate processing and data analysis methods included in our MIDAS software. Metabolic abnormalities were observed in PHIV subjects in both the GM and WM of the entire brain with evidence of strong lateralization in the left hemisphere of the brain being more heavily impacted than the right hemisphere. Most notable metabolic alterations were observed with tCre and tCho, both showing significantly elevated concentrations for PHIV in the GM of all four brain lobes, and in the WM of right frontal and parietal, and left temporal and occipital brain lobes. Inspection of specific anatomical ROIs from the AAL47 atlas shows these increases were mainly found in the frontal, pre-central, post-central, parietal, temporal lobes gyri, and the insula as well as in WM structures such as the cingulum and in basal ganglia structures such as the hippocampus, caudate, and putamen.

The tCho in the brain is typically considered as a biomarker of cellular synthesis, injury and degradation because tCho is required for major structural parts of neuronal and glial membrane phospholipids (e.g., phosphatidylcholine), synthesis of myelin sheath-associated compounds (e.g., sphingomyelin) which are important for axonal integrity, and synthesis of neurotransmitter acetylcholine (Ach) which is involved in sensory, motor, and cognitive functions (Govindaraju et al., 2000; Maurer and Williams, 2017; Li X. et al., 2018). Increases in MRS-observed tCho concentrations, as detected in the PHIV group, are reflective of cell membrane degradation, microglial proliferation, reactive astrogliosis, demyelination, and potential inflammation. The tCre, present in metabolically active tissues, plays a vital role in energy metabolism, storage, and transfer, and in the cytoprotection of most cells (Rackayova et al., 2017). *In vitro* studies have also shown that the creatine content of glial cells is two to four times that of neurons (Urenjak et al., 1993). Therefore, elevated tCre in PHIV can be interpreted as an increase in cell energy demand that compensate for increased cellular workload, glial and neuronal cell injuries, as well as a surrogate for active gliosis (Ratai et al., 2011). This is supported by studies showing a decline in tCre after HIV patients begin ART treatment (Chang et al., 2003). Between group differences in tNAA were less noticeable with only significant increases in the GM of the left parietal lobe for PHIV subjects because neurons are not directly infected by HIV but indirectly impacted by toxic HIV viral proteins in the brain extracellular environment. However, we note a trend of reduced tNAA in the WM and elevated tNAA in the GM from the lobar atlas, and reduced tNAA the lingual, fusiform, postcentral, and parietal gyri from the AAL47 atlas for PHIV subjects with significant uncorrected *p*-values (*p* < 0.05). These comparisons did not reach the significance level after FDR correction for multiple comparisons. Significant decreases were seen in tNAA/tCre and tNAA/tCho throughout the brain in both GM and WM tissue, but these alterations may be more driven by the elevated tCre and tCho. Therefore, we can conclude that subtle decreases in tNAA are found primarily in WM, indicating neuronal dysfunction or loss due to HIV infection. While some discrepancy has been found in the literature regarding brain metabolite changes in PHIV infected children, our findings are generally in concordance with most of the published research investigating brain metabolite in adults with behaviorally acquired HIV (Chelala et al., 2020).

Correlation analyses with clinical measures showed that tCre increased and tNAA/tCre decreased for subjects with lower CD4 cell count, respectively. Since tCre was higher and tNAA/tCre was lower among PHIV subjects, this shows that the severity of infection (indicated by lower CD4 count) was associated with poorer brain metabolic outcomes and more severe gliosis and neuronal loss. Associations with NP outcomes were comparatively less significant. We observed negative correlations between TRLS scores and tNAA in the GM of the right frontal lobe, the right frontal gyrus, and right anterior cingulum, and positive correlations between LET scores and tNAA/tCre in the WM of the left temporal and occipital lobes. In our previous study of cognitive outcomes from the same subject population (Willen et al., 2017), PHIV subjects performed more poorly on almost all NP variables including TRLS and LET tests. The increase in tNAA/tCre relative to increases in LET scores indicates that subjects with higher tNAA/tCre with improved neuronal function are performing better on LET tests. Conversely, TRLS scores are lower for subjects with higher tNAA, showing poorer test performance for subjects with better neuronal function. These associations are corroborated by structural imaging outcomes that found higher WM injury and smaller GM volumes correlating with higher viral load and worse cognitive performance (Uban et al., 2015; Lewis-de Los Angeles et al., 2017).

History of encephalopathy and meningitis did not have any effect on brain metabolites, as we had previously observed with respect to NP measures (Willen et al., 2017). This may be due to the long-elapsed time between those occurrences and data collection for this study.

### 4.1. Limitations

Despite the substantial findings, this study had few limitations including the small number of enrolled control participants, which was mitigated by the inclusion of age and gender matched control subjects from other studies conducted by our lab. This did not affect the processing and analysis of MRI data, as all subjects were scanned at the same facility with near identical MRI protocols that used the same whole-brain MRSI sequence. However, the additional control subjects could not be matched with the PHIV group by ethnicity. Another limitation was that we could not quantify myo-inositol (m-Ins) and glutamate/glutamine (Glx) because our MRSI acquisition was not optimized for capturing signals of m-Ins and Glx.

## 5. Conclusion

The results of this study show significant metabolic abnormalities in PHIV infected young adults relative to demographically matched controls at the whole-brain level, indicating increased glial activation and inflammation (higher tCho), neuronal dysfunction (lower tNAA and tNAA/tCre in WM), and increased energy metabolism (higher tCre). The degree of the alterations correlated with the severity of infection, with lower CD4 cell count observed among subjects exhibiting the highest brain metabolic alterations. These metabolic changes, along with neurocognitive deficits found in our previous study, suggest that PHIV infected children on long term ART continue to experience neurological and neurocognitive impairments as they transition into adulthood. While most of the PHIV subjects in question were closely following their ART regimens, a small yet not insignificant number of subjects had poor ART adherence that was reflected by the sub-par CD4 count observed in several subjects. This reinforces the fact that social, economic, and psychosocial stressors disproportionally impact HIV infected youth and may contribute to poorer adherence and subsequent sub-optimal virologic control. Our findings highlight the need for continued monitoring of health outcomes in PHIV infected youth, as they transition into adulthood, with targeted longitudinal studies.

## Data availability statement

The raw data supporting the conclusions of this article will be made available by the authors, without undue reservation.

## Ethics statement

The studies involving human participants were reviewed and approved by Institutional Review Board of University of Miami. The patients/participants provided their written informed consent to participate in this study.

## Author contributions

VG, EW, and AC designed the study. TS and VG analyzed the neuroimaging data. EW and AC recruited PHIV and some of the control subjects and analyzed the neurocognitive data. SS and AM provided the whole-brain EPSI data acquisition sequence and the MIDAS data analysis software and helped in the data processing. TS completed the final analysis and prepared the manuscript draft. All authors substantially contributed to the interpretation of data and manuscript revision and approved the submitted version.
