# Supplementary material for: Whole-brain MR spectroscopic imaging reveals regional metabolite abnormalities in perinatally HIV infected young adults
Source: Front Neurosci. 2023 Mar 2;17:1134867. doi: 10.3389/fnins.2023.1134867 (PMC10017464; doi:10.3389/fnins.2023.1134867)
Supplement: Supplementary file 1 [file Data_Sheet_1.docx]

Supplementary Material

Whole-brain MR Spectroscopic Imaging Reveals Regional Metabolite Abnormalities in Perinatally HIV Infected Young Adults

# Supplementary Tables

**Supplementary Table 1.** ROI abbreviations for the AAL47 atlas.

| ROI Name | Abbreviation |
| --- | --- |
| Pre-central | PrC |
| Frontal | Front |
| Rolandic Operculum | RolOper |
| Superior Motor Area | SMA |
| Insula | Ins |
| Anterior Cingulum | CingAnt |
| Middle Cingulum | CingMid |
| Posterior Cingulum | CingPost |
| Hippocampus | Hippo |
| Calcarine | Calc |
| Cuneus | Cu |
| Lingual | Ling |
| Occipital | Occ |
| Fusiform | Fu |
| Postcentral | PoC |
| Parietal | Par |
| Precuneus | PreCu |
| Paracentral lobule | PCL |
| Caudate | Caud |
| Putamen | Put |
| Pallidum | GP |
| Thalamus | Thal |
| Temporal | Temp |

**Supplementary Table 2.** ANCOVA results showing group mean, standard deviation (sd), and p-values for metabolite concentrations of tNAA, tCre, and tCho in all AAL47 atlas ROIs. ^#^ Values are (×10^3^) expressed in institutional units (i.u). * p < 0.05 (FDR corrected).

| ROI  (AAL47 Atlas) | tNAA | | | tCre | | | tCho | | |
| --- | --- | --- | --- | --- | --- | --- | --- | --- | --- |
|  | Control mean (sd)^#^ | PHIV  mean (sd)^#^ | *p* | Control mean (sd)^#^ | PHIV  mean (sd)^#^ | *p* | Control mean (sd)^#^ | PHIV  mean (sd)^#^ | *p* |
| PrC R | 14.48 (1.21) | 14.71 (1.41) | 0.925 | 8.75 (0.82) | 9.35 (0.72) | **0.024 *** | 1.8 (0.13) | 1.98 (0.22) | **0.003 *** |
| PrC L | 14.43 (1.23) | 14.95 (1.37) | 0.695 | 8.71 (0.88) | 9.52 (0.72) | **0.01 *** | 1.74 (0.21) | 1.97 (0.27) | **0.005 *** |
| Front R | 13.65 (1.21) | 13.28 (1.17) | 0.695 | 8.46 (0.78) | 8.82 (0.73) | **0.048 *** | 1.92 (0.17) | 2.02 (0.22) | 0.091 |
| Front L | 13.91 (0.73) | 13.79 (1) | 0.799 | 8.55 (0.66) | 9 (0.73) | **0.03 *** | 1.85 (0.17) | 1.99 (0.25) | **0.033 *** |
| RolOper R | 14.6 (0.88) | 14.56 (0.8) | 0.925 | 9.07 (0.71) | 9.61 (0.79) | **0.022 *** | 1.97 (0.21) | 2.05 (0.21) | 0.13 |
| RolOper L | 14.36 (0.72) | 14.59 (0.89) | 0.799 | 8.97 (0.72) | 9.69 (0.57) | **0.003 *** | 1.87 (0.22) | 2.05 (0.22) | **0.006 *** |
| SMA R | 13.6 (1.05) | 13.25 (0.99) | 0.436 | 8.79 (0.93) | 9.07 (0.84) | 0.427 | 1.99 (0.23) | 2.06 (0.34) | 0.555 |
| SMA L | 13.97 (1.14) | 13.93 (1.1) | 0.849 | 9.08 (0.99) | 9.74 (1.05) | **0.042 *** | 1.99 (0.23) | 2.13 (0.24) | 0.072 |
| Ins R | 15.34 (0.98) | 15.37 (0.86) | 1 | 10.06 (0.83) | 10.68 (0.85) | **0.013 *** | 2.54 (0.27) | 2.64 (0.27) | 0.15 |
| Ins L | 15.03 (0.77) | 15.17 (0.99) | 0.853 | 9.95 (0.81) | 10.62 (0.8) | **0.01 *** | 2.46 (0.29) | 2.62 (0.27) | **0.036 *** |
| CingAnt R | 14.66 (1.03) | 14.18 (1.25) | 0.505 | 9.87 (1.17) | 10.41 (1.05) | 0.089 | 2.72 (0.38) | 2.85 (0.41) | 0.264 |
| CingAnt L | 14.56 (1.26) | 14.63 (1.15) | 0.987 | 9.67 (1.15) | 10.38 (0.93) | **0.025 *** | 2.76 (0.39) | 2.89 (0.42) | 0.192 |
| CingMid R | 14.94 (0.8) | 15.12 (0.92) | 0.849 | 9.6 (0.64) | 10.07 (0.65) | **0.028 *** | 2.13 (0.21) | 2.29 (0.26) | **0.021 *** |
| CingMid L | 15.09 (0.85) | 15.42 (0.93) | 0.695 | 9.67 (0.63) | 10.27 (0.69) | **0.01 *** | 2.18 (0.22) | 2.32 (0.25) | **0.038 *** |
| CingPost R | 15.3 (1.19) | 15.17 (1.27) | 0.799 | 9.24 (0.74) | 9.49 (1.14) | 0.525 | 1.83 (0.17) | 1.92 (0.18) | 0.116 |
| CingPost L | 15.13 (1.24) | 15.16 (1.13) | 1 | 8.66 (1.05) | 9.04 (1.26) | 0.277 | 1.82 (0.27) | 1.89 (0.21) | 0.278 |
| Hippo R | 13.48 (1.27) | 13.28 (0.98) | 0.695 | 9.22 (1.18) | 9.84 (0.65) | **0.022 *** | 2.72 (0.26) | 2.93 (0.28) | **0.024 *** |
| Hippo L | 13.51 (1.13) | 13.34 (1.18) | 0.765 | 9.18 (1.05) | 9.77 (0.73) | **0.031 *** | 2.75 (0.24) | 2.88 (0.23) | 0.053 |
| Calc R | 15.57 (1.3) | 15.06 (0.99) | 0.375 | 9.69 (1.4) | 9.74 (0.87) | 0.417 | 1.57 (0.3) | 1.48 (0.21) | 0.384 |
| Calc L | 15.17 (1.07) | 14.98 (1.05) | 0.765 | 9.18 (1) | 9.57 (0.85) | **0.038 *** | 1.61 (0.2) | 1.6 (0.22) | 0.854 |
| Cu R | 14.86 (1.47) | 14.94 (1.24) | 0.987 | 9.08 (1.06) | 9.67 (1.14) | 0.087 | 1.41 (0.23) | 1.39 (0.24) | 0.813 |
| Cu L | 14.35 (1.03) | 14.58 (1.23) | 0.849 | 8.79 (0.82) | 9.4 (0.91) | **0.028 *** | 1.46 (0.21) | 1.47 (0.24) | 0.942 |
| Ling R | 15.27 (1.04) | 14.71 (0.91) | 0.207 | 10.44 (1.57) | 10.69 (1.21) | 0.395 | 1.88 (0.37) | 1.84 (0.28) | 0.813 |
| Ling L | 14.93 (1.04) | 14.74 (0.94) | 0.695 | 10.15 (1.43) | 10.77 (1.24) | **0.031 *** | 1.85 (0.28) | 1.88 (0.29) | 0.373 |
| Occ R | 14.72 (1.24) | 14.6 (0.83) | 0.765 | 8.77 (0.85) | 9.34 (0.91) | **0.047 *** | 1.52 (0.21) | 1.58 (0.22) | 0.451 |
| Occ L | 13.73 (1.08) | 14.24 (1.13) | 0.511 | 8.47 (0.75) | 9.21 (0.87) | **0.01 *** | 1.53 (0.21) | 1.6 (0.26) | 0.428 |
| Fu R | 15.08 (1.09) | 14.39 (1.28) | 0.219 | 10.64 (1.36) | 11.1 (1.39) | 0.258 | 2.3 (0.36) | 2.36 (0.19) | 0.486 |
| Fu L | 14.95 (1.17) | 14.08 (1.32) | 0.207 | 10.57 (1.56) | 10.82 (1.5) | 0.555 | 2.29 (0.21) | 2.31 (0.2) | 0.794 |
| PoC R | 14.55 (0.93) | 14.5 (1.04) | 0.799 | 9.02 (0.79) | 9.53 (0.75) | **0.041 *** | 1.73 (0.18) | 1.89 (0.22) | **0.019 *** |
| PoC L | 13.9 (0.97) | 14.64 (1.2) | 0.222 | 8.76 (0.69) | 9.65 (0.67) | **0.001 *** | 1.62 (0.18) | 1.96 (0.26) | **<0.001*** |
| Par R | 14.65 (0.9) | 15.3 (0.97) | 0.219 | 9.07 (0.8) | 9.93 (0.84) | **0.004 *** | 1.69 (0.18) | 1.94 (0.21) | **<0.001*** |
| Par L | 14.16 (0.96) | 15.02 (1.24) | 0.207 | 8.92 (0.71) | 9.84 (0.73) | **0.001 *** | 1.65 (0.16) | 1.91 (0.26) | **<0.001*** |
| PreCu R | 15.02 (0.74) | 15.11 (0.74) | 1 | 9.59 (0.73) | 10.23 (0.86) | **0.025 *** | 1.7 (0.15) | 1.78 (0.18) | 0.094 |
| PreCu L | 14.79 (0.69) | 15.02 (0.87) | 0.799 | 9.27 (0.73) | 9.9 (0.76) | **0.01 *** | 1.71 (0.15) | 1.83 (0.19) | **0.021 *** |
| PCL R | 13.97 (1.17) | 14.15 (1.29) | 0.965 | 9.25 (1.09) | 9.86 (1.1) | 0.091 | 1.86 (0.28) | 1.93 (0.28) | 0.574 |
| PCL L | 13.66 (1.24) | 14.4 (1.32) | 0.375 | 9.32 (0.95) | 10.14 (1.27) | **0.028 *** | 1.78 (0.19) | 2 (0.24) | **0.002 *** |
| Caud R | 14.05 (1.17) | 13.76 (1.03) | 0.695 | 8.89 (0.76) | 9.62 (0.93) | **0.011 *** | 2.55 (0.25) | 2.7 (0.31) | **0.036 *** |
| Caud L | 14.46 (1.14) | 14.04 (1.03) | 0.436 | 9.03 (0.96) | 9.71 (0.93) | **0.025 *** | 2.57 (0.31) | 2.78 (0.37) | **0.027 *** |
| Put R | 14.33 (1.15) | 14.15 (1.11) | 0.799 | 9.69 (0.83) | 10.38 (0.69) | **0.01 *** | 2.34 (0.25) | 2.49 (0.26) | 0.053 |
| Put L | 14.39 (0.93) | 14.17 (0.93) | 0.695 | 9.61 (0.89) | 10.45 (0.79) | **0.008 *** | 2.4 (0.23) | 2.53 (0.23) | 0.051 |
| GP R | 14.13 (1.51) | 13.66 (1.47) | 0.695 | 9.2 (0.67) | 9.46 (0.79) | 0.247 | 2.18 (0.31) | 2.34 (0.34) | 0.116 |
| GP L | 13.74 (1.4) | 13.49 (1.13) | 0.799 | 9.26 (1.03) | 9.77 (1.15) | 0.159 | 2.14 (0.25) | 2.33 (0.32) | 0.174 |
| Thal R | 14.51 (1.29) | 14.42 (1.31) | 0.799 | 9.21 (0.76) | 9.69 (0.81) | 0.052 | 2.66 (0.26) | 2.79 (0.31) | 0.089 |
| Thal L | 14.72 (1.35) | 14.83 (1.4) | 1 | 9.32 (0.96) | 9.84 (0.79) | 0.051 | 2.65 (0.29) | 2.86 (0.23) | **0.006 *** |
| Temp R | 14.6 (1.02) | 14.33 (1.13) | 0.695 | 9.22 (0.81) | 9.79 (1.12) | **0.044 *** | 1.97 (0.25) | 2.14 (0.27) | **0.034 *** |
| Temp L | 14.62 (0.99) | 14.53 (1.11) | 0.911 | 9.26 (0.71) | 9.93 (0.93) | **0.01 *** | 1.92 (0.19) | 2.11 (0.23) | **0.006 *** |

**Supplementary Table 3.** ANCOVA results showing group mean, standard deviation (sd), and p-values for metabolite concentrations of tNAA/tCre, tCho/tCre, and tNAA/tCho in all AAL47 atlas ROIs. ^#^ Values are (×10^3^) expressed in institutional units (i.u). * p < 0.05 (FDR corrected).

| ROI  (AAL47 Atlas) | tNAA/tCre | | | tCho/tCre | | | tNAA/tCho | | |
| --- | --- | --- | --- | --- | --- | --- | --- | --- | --- |
|  | Control mean (sd)^#^ | PHIV  mean (sd)^#^ | *p* | Control mean (sd)^#^ | PHIV  mean (sd)^#^ | *p* | Control mean (sd)^#^ | PHIV  mean (sd)^#^ | *p* |
| PrC R | 1.65 (0.12) | 1.56 (0.13) | **0.006 *** | 0.21 (0.02) | 0.21 (0.02) | 0.995 | 8.39 (1.01) | 7.44 (0.86) | **0.005 *** |
| PrC L | 1.68 (0.14) | 1.56 (0.15) | **0.006 *** | 0.2 (0.02) | 0.21 (0.03) | 0.995 | 8.84 (1.33) | 7.92 (1.03) | **0.012 *** |
| Front R | 1.61 (0.1) | 1.49 (0.15) | **0.002 *** | 0.23 (0.02) | 0.22 (0.02) | 0.725 | 7.45 (0.89) | 7.06 (1.35) | 0.061 |
| Front L | 1.64 (0.12) | 1.56 (0.1) | **0.021 *** | 0.22 (0.03) | 0.22 (0.02) | 0.997 | 8.26 (0.94) | 7.51 (1.15) | **0.012 *** |
| RolOper R | 1.61 (0.13) | 1.48 (0.14) | **0.002 *** | 0.22 (0.02) | 0.21 (0.02) | 0.725 | 7.62 (0.85) | 7.16 (0.97) | 0.067 |
| RolOper L | 1.62 (0.14) | 1.5 (0.13) | **0.002 *** | 0.21 (0.02) | 0.21 (0.02) | 0.995 | 7.93 (0.88) | 7.32 (0.81) | **0.012 *** |
| SMA R | 1.59 (0.17) | 1.49 (0.15) | **0.034 *** | 0.23 (0.02) | 0.23 (0.04) | 0.995 | 7.28 (1.06) | 6.96 (1.16) | 0.136 |
| SMA L | 1.56 (0.15) | 1.46 (0.15) | **0.018 *** | 0.22 (0.03) | 0.22 (0.02) | 0.995 | 7.43 (0.93) | 6.97 (0.92) | 0.067 |
| Ins R | 1.57 (0.14) | 1.46 (0.12) | **0.005 *** | 0.26 (0.02) | 0.25 (0.02) | 0.725 | 6.31 (0.79) | 6.01 (0.67) | 0.12 |
| Ins L | 1.55 (0.13) | 1.45 (0.13) | **0.009 *** | 0.25 (0.02) | 0.25 (0.03) | 0.995 | 6.37 (0.72) | 5.97 (0.68) | **0.025 *** |
| CingAnt R | 1.56 (0.18) | 1.44 (0.22) | **0.034 *** | 0.3 (0.05) | 0.28 (0.04) | 0.725 | 5.84 (1.13) | 5.49 (0.91) | 0.369 |
| CingAnt L | 1.62 (0.23) | 1.57 (0.32) | **0.033 *** | 0.31 (0.06) | 0.3 (0.05) | 0.725 | 6.31 (1.32) | 5.89 (1.56) | 0.061 |
| CingMid R | 1.58 (0.13) | 1.52 (0.11) | **0.049 *** | 0.23 (0.02) | 0.23 (0.03) | 0.995 | 7.39 (0.78) | 6.96 (0.89) | 0.059 |
| CingMid L | 1.59 (0.14) | 1.53 (0.12) | 0.059 | 0.23 (0.03) | 0.23 (0.03) | 0.995 | 7.51 (1.09) | 7.04 (0.84) | 0.12 |
| CingPost R | 1.7 (0.2) | 1.62 (0.23) | 0.199 | 0.21 (0.02) | 0.2 (0.03) | 0.997 | 8.4 (0.93) | 8.06 (0.97) | 0.196 |
| CingPost L | 1.82 (0.25) | 1.72 (0.28) | 0.244 | 0.22 (0.03) | 0.21 (0.03) | 0.995 | 8.56 (1.2) | 8.23 (1.07) | 0.279 |
| Hippo R | 1.5 (0.16) | 1.37 (0.1) | **0.003 *** | 0.3 (0.03) | 0.3 (0.03) | 0.997 | 5.05 (0.44) | 4.65 (0.54) | **0.012 *** |
| Hippo L | 1.51 (0.16) | 1.37 (0.13) | **0.002 *** | 0.3 (0.03) | 0.29 (0.03) | 0.725 | 5.1 (0.52) | 4.73 (0.53) | **0.012 *** |
| Calc R | 1.68 (0.13) | 1.57 (0.13) | **0.006 *** | 0.17 (0.02) | 0.16 (0.02) | 0.725 | 10.92 (1.74) | 10.91 (1.6) | 0.851 |
| Calc L | 1.68 (0.16) | 1.58 (0.15) | **0.008 *** | 0.18 (0.02) | 0.17 (0.02) | 0.725 | 10.13 (1.52) | 9.97 (1.43) | 0.592 |
| Cu R | 1.63 (0.12) | 1.55 (0.14) | **0.032 *** | 0.15 (0.02) | 0.14 (0.02) | 0.725 | 11.31 (1.84) | 11.47 (1.74) | 0.878 |
| Cu L | 1.64 (0.15) | 1.57 (0.14) | **0.049 *** | 0.17 (0.02) | 0.16 (0.02) | 0.725 | 10.35 (1.55) | 10.42 (1.57) | 0.983 |
| Ling R | 1.49 (0.17) | 1.38 (0.17) | **0.034 *** | 0.18 (0.02) | 0.17 (0.02) | 0.725 | 8.82 (1.55) | 8.52 (1.6) | 0.562 |
| Ling L | 1.5 (0.2) | 1.38 (0.17) | **0.029 *** | 0.18 (0.02) | 0.18 (0.02) | 0.725 | 8.71 (1.44) | 8.35 (1.53) | 0.41 |
| Occ R | 1.62 (0.13) | 1.51 (0.11) | **0.005 *** | 0.17 (0.02) | 0.16 (0.02) | 0.965 | 9.87 (1.45) | 9.36 (1.27) | 0.271 |
| Occ L | 1.62 (0.14) | 1.56 (0.14) | 0.102 | 0.18 (0.03) | 0.17 (0.02) | 0.762 | 9.31 (1.33) | 9.47 (1.44) | 0.755 |
| Fu R | 1.41 (0.14) | 1.32 (0.15) | **0.034 *** | 0.21 (0.03) | 0.21 (0.02) | 0.965 | 6.85 (0.92) | 6.35 (0.69) | 0.061 |
| Fu L | 1.48 (0.19) | 1.33 (0.15) | **0.005 *** | 0.23 (0.03) | 0.22 (0.03) | 0.965 | 6.84 (0.69) | 6.3 (0.74) | **0.017 *** |
| PoC R | 1.59 (0.11) | 1.46 (0.13) | **0.002 *** | 0.19 (0.02) | 0.19 (0.02) | 0.995 | 8.51 (1.01) | 7.62 (0.92) | **0.006 *** |
| PoC L | 1.59 (0.14) | 1.51 (0.13) | **0.009 *** | 0.19 (0.02) | 0.2 (0.03) | 0.725 | 8.81 (1.21) | 7.81 (1.2) | **<0.001*** |
| Par R | 1.57 (0.12) | 1.51 (0.11) | **0.042 *** | 0.18 (0.02) | 0.19 (0.02) | 0.965 | 8.54 (0.97) | 7.86 (1.01) | **0.018 *** |
| Par L | 1.6 (0.14) | 1.53 (0.12) | **0.034 *** | 0.19 (0.02) | 0.19 (0.02) | 0.725 | 8.72 (0.74) | 8.11 (1.2) | **0.025 *** |
| PreCu R | 1.57 (0.11) | 1.5 (0.13) | **0.023 *** | 0.18 (0.02) | 0.18 (0.02) | 0.995 | 9.1 (0.86) | 8.88 (0.99) | 0.27 |
| PreCu L | 1.62 (0.13) | 1.54 (0.11) | **0.018 *** | 0.19 (0.02) | 0.19 (0.02) | 0.995 | 8.96 (0.89) | 8.52 (1.04) | 0.067 |
| PCL R | 1.53 (0.14) | 1.46 (0.16) | 0.116 | 0.2 (0.03) | 0.2 (0.03) | 0.965 | 7.95 (1.55) | 7.67 (1.03) | 0.755 |
| PCL L | 1.46 (0.11) | 1.43 (0.15) | 0.256 | 0.2 (0.03) | 0.2 (0.03) | 0.725 | 8.01 (1.14) | 7.33 (1.04) | **0.036 *** |
| Caud R | 1.61 (0.15) | 1.48 (0.16) | **0.006 *** | 0.29 (0.03) | 0.29 (0.03) | 0.997 | 5.84 (0.84) | 5.29 (0.78) | **0.012 *** |
| Caud L | 1.63 (0.16) | 1.48 (0.2) | **0.011 *** | 0.29 (0.04) | 0.29 (0.05) | 0.995 | 5.85 (0.83) | 5.25 (0.82) | **0.012 *** |
| Put R | 1.5 (0.14) | 1.38 (0.1) | **0.004 *** | 0.25 (0.02) | 0.24 (0.02) | 0.995 | 6.41 (1.39) | 5.96 (0.9) | 0.061 |
| Put L | 1.51 (0.13) | 1.38 (0.11) | **0.002 *** | 0.25 (0.03) | 0.25 (0.02) | 0.725 | 6.13 (0.62) | 5.83 (0.77) | **0.023 *** |
| GP R | 1.56 (0.2) | 1.47 (0.19) | 0.12 | 0.24 (0.03) | 0.25 (0.04) | 0.725 | 6.7 (1.07) | 6.03 (0.77) | **0.023 *** |
| GP L | 1.5 (0.2) | 1.41 (0.16) | **0.049 *** | 0.23 (0.03) | 0.24 (0.02) | 0.725 | 6.51 (0.69) | 5.97 (0.71) | **0.023 *** |
| Thal R | 1.6 (0.13) | 1.51 (0.13) | **0.01 *** | 0.29 (0.03) | 0.29 (0.03) | 0.997 | 5.58 (0.55) | 5.24 (0.62) | **0.017 *** |
| Thal L | 1.63 (0.17) | 1.53 (0.15) | **0.016 *** | 0.29 (0.03) | 0.29 (0.03) | 0.965 | 5.69 (0.68) | 5.28 (0.6) | **0.017 *** |
| Temp R | 1.54 (0.11) | 1.4 (0.13) | **0.002 *** | 0.21 (0.02) | 0.21 (0.02) | 0.997 | 7.62 (0.96) | 6.75 (1.06) | **0.012 *** |
| Temp L | 1.6 (0.13) | 1.47 (0.16) | **0.006 *** | 0.21 (0.02) | 0.21 (0.02) | 0.965 | 7.95 (0.77) | 7.23 (1.03) | **0.019 *** |
